# Learning Invariant Object and Spatial View Representations in the Brain Using Slow Unsupervised Learning

**DOI:** 10.3389/fncom.2021.686239

**Published:** 2021-07-21

**Authors:** Edmund T. Rolls

**Affiliations:** ^1^Oxford Centre for Computational Neuroscience, Oxford, United Kingdom; ^2^Department of Computer Science, University of Warwick, Coventry, United Kingdom

**Keywords:** face cells, spatial view cells, hippocampus, navigation, object recognition, inferior temporal visual cortex, unsupervised learning, convolutional neural network

## Abstract

First, neurophysiological evidence for the learning of invariant representations in the inferior temporal visual cortex is described. This includes object and face representations with invariance for position, size, lighting, view and morphological transforms in the temporal lobe visual cortex; global object motion in the cortex in the superior temporal sulcus; and spatial view representations in the hippocampus that are invariant with respect to eye position, head direction, and place. Second, computational mechanisms that enable the brain to learn these invariant representations are proposed. For the ventral visual system, one key adaptation is the use of information available in the statistics of the environment in slow unsupervised learning to learn transform-invariant representations of objects. This contrasts with deep supervised learning in artificial neural networks, which uses training with thousands of exemplars forced into different categories by neuronal teachers. Similar slow learning principles apply to the learning of global object motion in the dorsal visual system leading to the cortex in the superior temporal sulcus. The learning rule that has been explored in VisNet is an associative rule with a short-term memory trace. The feed-forward architecture has four stages, with convergence from stage to stage. This type of slow learning is implemented in the brain in hierarchically organized competitive neuronal networks with convergence from stage to stage, with only 4-5 stages in the hierarchy. Slow learning is also shown to help the learning of coordinate transforms using gain modulation in the dorsal visual system extending into the parietal cortex and retrosplenial cortex. Representations are learned that are in allocentric spatial view coordinates of locations in the world and that are independent of eye position, head direction, and the place where the individual is located. This enables hippocampal spatial view cells to use idiothetic, self-motion, signals for navigation when the view details are obscured for short periods.

## Introduction

This paper describes advances in how slow learning that takes advantage of the statistics of the environment is a useful principle in helping to build not only invariant representations in the ventral visual system of objects and faces, and invariant representations of object-based motion, but also allocentric spatial view representations in the parietal cortex and posterior cingulate cortex for use by the hippocampus in memory and navigation. The principles of slow learning described here may enable some transform-invariant representations to be learned that are not possible using principles of symmetry alone.

First neurophysiological evidence on the transform-invariant neuronal representations that are found in the primate ventral visual system is described. Then a biologically plausible approach, VisNet, to how unsupervised learning in hierarchical feedforward networks is computed, is updated with recent research. VisNet is a 4-Layer hierarchical network with convergence from stage to stage that emulates the architecture of the primate ventral visual system. Each stage operates as a competitive network, and uses slow learning with an associative synaptic modification rule to learn from the statistics of the environment. In the short term, the statistics tend to be about the same object etc., because of the way in which visual objects are fixated for short periods, during which different transforms may be shown (Rolls, 2012, 2021a). This contrasts with deep supervised learning in artificial neural networks, which uses training with thousands of exemplars forced into different categories by neuronal teachers using backpropagation of error learning (LeCun et al., 2010; LeCun et al., 2015; Yamins and DiCarlo, 2016). Key aspects of VisNet that are described as biologically plausible are that it uses a local synaptic learning rule in which the information is present in the pre- and postsynaptic rates without the need for backpropagation of error as in deep learning (LeCun et al., 2010, 2015; Yamins and DiCarlo, 2016) or for lateral propagation of synaptic weights as in deep convolution networks (Yamins and DiCarlo, 2016; Rajalingham et al., 2018); that it is unsupervised and self-organizing, without any need for a teacher for every output neuron; and that it learns by using information present in the statistics of the inputs from the natural world that tend to be about the same object, spatial view, etc. over short periods and that can be utilized by slow learning. These properties make VisNet an important model for the learning of invariant representations in the brain; and of interest for the development of future unsupervised artificial neural networks by providing some guiding principles.

This biologically plausible approach to transform-invariant object recognition was initiated by Rolls (1992), which was followed by a full model (Wallis and Rolls, 1997), and an updated description with many results (Rolls, 2012). The present paper provides an update to Rolls (2012) on the architecture and further developments with VisNet, together with a description of a version of VisNet written in Matlab for tutorial use (Rolls, 2021a). The further developments include finding and recognizing objects in natural scenes using saliency in the dorsal visual system to fixate on objects, combined with invariant object recognition in the ventral visual system to recognize the object at the fixated location (Rolls and Webb, 2014). Further developments are how non-accidental properties of objects can be learned by the slow learning implemented in VisNet (Rolls and Mills, 2018); and how visually different views of objects can be recognized as of the same object by VisNet but not by HMAX (Robinson and Rolls, 2015). This paper also extends this unsupervised learning approach to object-based motion in the dorsal visual system (Rolls and Stringer, 2006b) to provide a mechanism for the object-based motion representations found in the cortex in the superior temporal sulcus (Hasselmo et al., 1989b).

This paper also extends this slow learning approach to coordinate transforms in the dorsal visual system and parietal cortex that result in allocentric (world based) coordinates (Rolls, 2020), and that allow hippocampal spatial view cells to be updated by self-motion for navigation when the view is temporarily obscured (Rolls, 2021b). This extension helps to show how the slow learning approach used in VisNet that uses statistics present from the environmental inputs may be useful in a number of different brain systems.

The present paper also contrasts the unsupervised slow learning implemented in VisNet with many current approaches to vision that use deep learning and convolutional networks, highlighting what needs to be incorporated into models that may apply to understanding the brain, and some principles that are likely to be useful in future developments of artificial neural networks.

## Transform-Invariant Representations of Objects and Faces

### Neuronal Responses in the Brain With Transform-Invariant Responses to Objects and Faces

While recording in the inferior temporal visual cortex and amygdala, we discovered face cells, which respond in macaques much more to the sight of faces than to non-face visual stimuli (Perrett et al., 1979, 1982; Sanghera et al., 1979; Rolls, 1984, 2011, 2012, 2021a), with consistent findings by others (Desimone et al., 1984; Desimone, 1991; Gross, 1992; Sheinberg and Logothetis, 2001; Freiwald and Tsao, 2010; Li and DiCarlo, 2012; Tsao, 2014).

Many properties were discovered, including translation (Tovee et al., 1994), size and contrast (Rolls and Baylis, 1986), lighting (Rolls and Stringer, 2006a), spatial frequency (Rolls et al., 1987), and even for some neurons view (Hasselmo et al., 1989b), invariance; sparse distributed tuning to different faces (Rolls and Tovee, 1995; Rolls et al., 1997b, c; Franco et al., 2007; Rolls and Treves, 2011); the sensitivity of these neurons to combinations of features in the correct spatial arrangement (Perrett et al., 1979; Rolls et al., 1994); and the tuning for some neurons to face identity, and of others to face expression, and face and head motion (Rolls et al., 1987; Hasselmo et al., 1989a, b; Rolls, 2012, 2021a). All of these properties make them useful for natural behavior, because as a population they encode the identity of an individual in an invariant way, so that when associated with an outcome (for example a social reward, or punisher) in the next brain region, there would be automatic generalization of the association learning to other transformed views of the same individual or object (Rolls, 2021a).

Similar neurons in the inferior temporal visual cortex code in a transform-invariant way for objects (Booth and Rolls, 1998) including in natural scenes, and use sparse distributed firing rate coding not temporal coding (Rolls et al., 2003, 2006a; Aggelopoulos et al., 2005; Franco et al., 2007; Rolls and Treves, 2011; Rolls, 2021a).

A key property of inferior temporal cortex neurons that code for objects or faces that is relevant to how the brain recognizes objects is that their receptive fields shrink to about the size of objects in complex natural scenes, so that the whole scene is not computed at one time, but instead there are repeated fixations to different parts of a scene, with object recognition performed separately for each part of a scene (Rolls et al., 2003; Aggelopoulos and Rolls, 2005; Aggelopoulos et al., 2005; Rolls, 2021a).

Another key property of these neurons for understanding the mechanisms of visual perception is that they can perform visual object recognition using forward processing only without backward propagation of any signals being important, as shown by experiments with backward visual masking (Rolls and Tovee, 1994; Rolls et al., 1999; Rolls, 2003, 2005, 2021a).

Another important property is that these inferior temporal cortex neurons modify their responses to new but not already familiar objects in the first few presentations of a new object (Rolls et al., 1989a; Tovee et al., 1996; Dolan et al., 1997), providing evidence on how new representations are built by a self-organizing process in the temporal lobe cortex.

All of these neuronal response properties of macaque inferior temporal cortex and related neurons described more fully elsewhere (Rolls, 2021a) were used to help design the model of invariant visual object recognition, VisNet, described next, which has, as a key goal, helping to understand the mechanisms of visual object and face perception in the primate including human brain.

### Unsupervised Slow Learning of Transform-Invariant Representations in a Model of the Ventral Visual System, VisNet

#### The Architecture of VisNet

**FIGURE 1:**
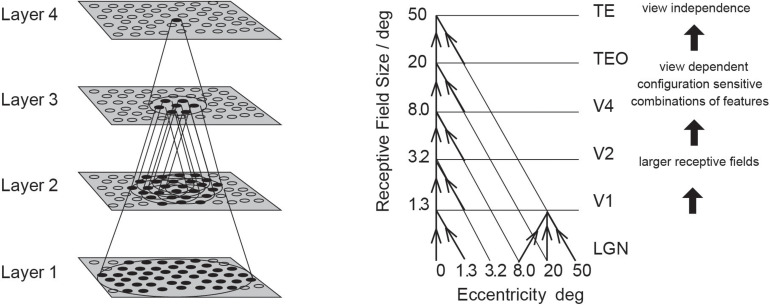
Convergence in the visual system. **(Right)** Convergence in the ventral stream cortical hierarchy for object recognition. LGN, lateral geniculate nucleus; V1, visual cortex area V1; TEO, posterior inferior temporal cortex; TE, anterior inferior temporal cortex (IT). **(Left)** Convergence as implemented in VisNet, the model of invariant visual object recognition described here. Convergence through the hierarchical feedforward network is designed to provide Layer 4 neurons with information from across the entire input retina, by providing an increase of receptive field size of 2.5 times at each stage. Layer 1 of the VisNet model corresponds to V2 in the brain, and Layer 4 to the anterior inferior temporal visual cortex (TE). In this paper ‘Layer’ with a capital L indicates a Layer of a neuronal network which may correspond to a brain region as here. This is distinct from the 6 architectonic layers in neocortex, designated here with a small letter l in ‘layer’.

Having discovered many properties of inferior temporal cortex neurons, Rolls was keen to go beyond phenomenology to mechanisms that might produce such interesting neurons (Rolls, 1992). He proposed that hierarchical organization from V1 via V2 and V4 to the inferior temporal visual cortex with convergence from stage to stage and competitive learning was a way to set up neurons with large receptive fields that could become tuned to feature combinations that represent objects, and do this with translation invariance (Figure 1). VisNet is a feature hierarchy network [described in detail elsewhere) (Rolls, 2016, 2021a)], and emulates to some extent the sparse distributed encoding that is found for objects and faces in the ventral visual system (Rolls and Treves, 2011; Rolls, 2021a). The hierarchical organization is important for brain systems to learn about the natural world, because it means that a single neuron need receive only a limited number (∼10,000) inputs from the previous stage (Figure 1). Important aspects of the design to make it biologically plausible is that the whole problem is solved in a network with only four Layers; that the computation is feedforward, with no feedback of errors or anything else required for learning; and with no supervision of the training by for example separate teachers for each neuron in the output Layer.

##### The short-term memory trace learning rule used in VisNet

A key part of the proposal for VisNet is learning that uses a short-term memory trace for previous neuronal activity, so that the neurons could learn to respond to different transforms of an object, which in the real world typically occur close together in time (Rolls, 1992). A similar principle had been proposed for translation invariance (Földiák, 1991), but Rolls extended this to all types of invariance, and outlined how this could be set up in a hierarchical model (Rolls, 1992). The full model was built (Wallis et al., 1993; Wallis and Rolls, 1997), which is known as VisNet (Rolls, 2012), and a reduced version of which in Matlab is available with *Brain Computations: What and How* (Rolls, 2021a). The trace learning rule is biologically plausible, and could involve processes such as the long time constant of NMDA receptors, or local cortical attractor network operations, which do keep cortical neurons firing for a few hundred ms (Rolls and Tovee, 1994; Rolls, 2003, 2021a).

The short-term memory trace that enables inputs occurring close together in time, as they would in the natural world, to become associated is implemented in the hierarchical competitive network (Rolls, 2012, 2021a) model by using associative synaptic modification with a small change that allows the postsynaptic term to remain active for short periods in the order of 100 ms or more. The short-term memory trace update learning rule that we have used has the following form (Rolls, 2012, 2021a):

(1) $$\delta w_{\text{j}}=\alpha {\bar{y}}^{\tau }x_{\text{j}}$$

where

(2) $${\bar{y}}^{\tau }=\left(1-\eta \right)y^{\tau }+\eta {\bar{y}}^{\tau -1}$$

and

*x*_*j*_ is the *j*^*th*^ input to the neuron;

*y* is the output from the neuron;

${\bar{y}}^{\tau }$: is the Trace value of the output of the neuron at time step *τ*;

α is the learning rate;

*w*_*j*_ is the synaptic weight between the *j*^*th*^ input and the neuron;

*η* is the trace update proportion, with 0 meaning no trace, just associative learning. The optimal value varies with the number of transforms of each object, and is typically 0.8. Many variations of this learning rule have been explored (Rolls and Milward, 2000; Rolls and Stringer, 2001). The general form of the rule for computational purposes can be as shown in Equation (1), but the actual mechanism in the brain might utilize a slow synaptic eligibility trace such as provided by the NMDA receptors with their long time constant, as well as a tendency for neuronal firing to continue due to local attractor networks (Rolls, 2012, 2021a).

During training, all transforms of one object are presented in random sequence so that the trace rule can help learning that all of these are transforms of the same object because they occur close together in time; then all transforms of another object are shown; etc.

Layer 1 of VisNet is trained with a purely associative learning rule with no short-term memory trace, to enable feature combination neurons to be formed that represent the relative spatial locations of the features before any invariance learning starts in Layer 2. This solves the feature binding problem, as described below and elsewhere (Rolls, 2012, 2021a).

##### The VisNet network

VisNet consists of a series of feedforward hierarchically connected competitive networks with convergence from Layer to Layer, with four Layers, as illustrated in Figure 1. The connections to a neuron in one Layer come from a confined and topologically related region of the preceding Layer. The connections to a neuron in one Layer come from a small region of the preceding Layer using a Gaussian distribution of connection probabilities defined by the radius which will contain approximately 67% of the connections from the preceding Layer. Table 1 shows this radius for each Layer of 32 × 32 neurons per Layer, with each neuron receiving 200 synaptic connections from the neurons in the preceding Layer. The radii are set so that neurons at the fourth Layer of VisNet are able to be influenced by inputs from a stimulus at any location in Layer 1 (Rolls, 2012). The activation of a neuron is calculated as the synaptically weighted sum of the rate inputs it receives from the preceding Layer, i.e., as a dot or inner product between the input rates and the synaptic weights (Rolls and Milward, 2000; Rolls, 2012, 2021a; Rolls and Mills, 2018). The activations are converted into rates with a sigmoid or threshold-linear activation function, with the sparseness of the representation in a Layer set as described next.

**TABLE 1:** VisNet dimensions.

	Dimensions	# connections	Radius
Layer 4	32 × 32	200	12
Layer 3	32 × 32	200	12
Layer 2	32 × 32	200	12
Layer 1	32 × 32	272	15
Input layer	256 × 256 × 32	–	–

*Dimensions shows the number of neurons in each of the 4 Layers. # Connections shows the number of synaptic connections onto each neuron. Radius shows the radius of the connectivity from the previous Layer of a single neuron (see text). This is for the small tutorial version of VisNet written in Matlab and made available with Brain Computations: What and How (Rolls, 2021a). That tutorial version of VisNet can be scaled up to at least 256 × 256 neurons per Layer, and 1,000 synaptic connections to each neuron.*

##### Competition and mutual inhibition in VisNet

In a competitive network (Rolls, 2021a), mutual inhibition is required between the neurons within each Layer, so that for any one stimulus only a proportion of neurons is active. The activation of the neurons in a Layer is first calculated by the dot product of the synaptic weights of a neuron and the rates of the neurons in the preceding Layer to which it is connected by the synaptic weights. Then the activations are converted into rates using a sigmoid or threshold linear activation function, and the threshold for the activation function is set so that the sparseness across the neurons of the rates becomes a value specified by a sparseness parameter *a* that is typically 0.01, where sparseness is defined as

(3) $$a=\frac{{\left(\sum_{i}y_{\text{i}}/n\right)}^{2}}{\sum_{i}y_{\text{i}}^{2}/n}$$

where *n* is the number of neurons in the Layer, and *y*_*i*_ is the firing rate of the *i*th neuron in a Layer. Setting the sparseness in this way implements a form of competition within the network, in that only the neurons with the highest activations have rates greater than zero after the sparseness has been set as specified. This measure of sparseness is one that is useful in the quantitative analysis of the capacity of neuronal networks (Rolls and Treves, 1990; Treves, 1991; Treves and Rolls, 1991; Rolls, 2016, 2021a), and in neurophysiological measures of neuronal representations in the brain (Rolls and Tovee, 1995; Franco et al., 2007; Rolls and Treves, 2011; Rolls, 2016, 2021a). If the neurons have binary rates, the sparseness is the proportion of neurons that is active for any one stimulus.

##### The inputs to VisNet are provided by V1-like neurons produced by Gabor filtering of input images

The inputs to VisNet are computed to have elongated receptive fields of the type found in the primary visual cortex V1, in order to allow comparison of the neurons in VisNet at different stages to those in the brain. The Gabor filters (Daugman, 1988) have four spatial frequencies, four orientations, and positive or negative. The Layer one neurons are connected to these with radii as described above and in Table 1, and with the number of connections to each frequency scaled according to the spatial frequency, as described in detail elsewhere (Rolls, 2012, 2021a; Rolls and Mills, 2018).

#### Different Learning Rules in VisNet

The learning rule used in the upper Layers of VisNet to perform transform-invariant learning is by default purely associative learning involving a post-synaptic trace of recent neuronal activity and the presynaptic rate input (Eqn. 1), as this is very biologically plausible (Wallis and Rolls, 1997; Rolls and Milward, 2000). More powerful learning rules that use local (not back-propagated) error correction learning or local temporal difference learning have been investigated, and these can improve the learning of transform-invariant representations considerably (Rolls and Stringer, 2001). They all involve information that is potentially local, that is present at the synapse, and do not require an external teacher to provide the training signal for a particular neuron or synapse.

A very simple example of a rule of this type involves increasing the synaptic weights of active inputs if the short-term memory trace ${\bar{y}}^{\tau }$ is greater than the current firing *y*; and decreasing the synaptic weights of active inputs if the short term memory trace ${\bar{y}}^{\tau }$ is less than the current firing *y*, as follows:

(4) $$\delta w_{\text{j}}=\alpha \left({\bar{y}}^{\tau }-y\right)x_{\text{j}}$$

This version of the learning rule is available with the Matlab version of VisNet made available with *Brain Computations: What and How* (Rolls, 2021a). Many more types of learning rule are described by Rolls and Stringer (2001).

#### Translation and View Invariant Representations

This trace rule learning has been shown to be useful as a key principle of training of biologically plausible models of learning translation, size, and view invariant representations of objects and faces (Wallis and Rolls, 1997; Stringer and Rolls, 2000, 2002, 2008; Rolls, 2012, 2016, 2021a; Perry et al., 2006; Rolls and Webb, 2014).

#### Feature Binding

VisNet is a feature hierarchy network, which forms feature combination neurons at each stage of the network using competitive learning (Rolls, 2021a). It is important that features are bound together early on in processing in the correct relative spatial position. For example, a vertical and horizontal line might form a T, or an L, or a +. To ensure that the relative spatial positions of features are learned before any invariance is learned which would destroy the feature binding just described, the first Layer of VisNet (corresponding to V2) uses purely associative learning, without any temporal trace of previous activity.

**FIGURE 2:**
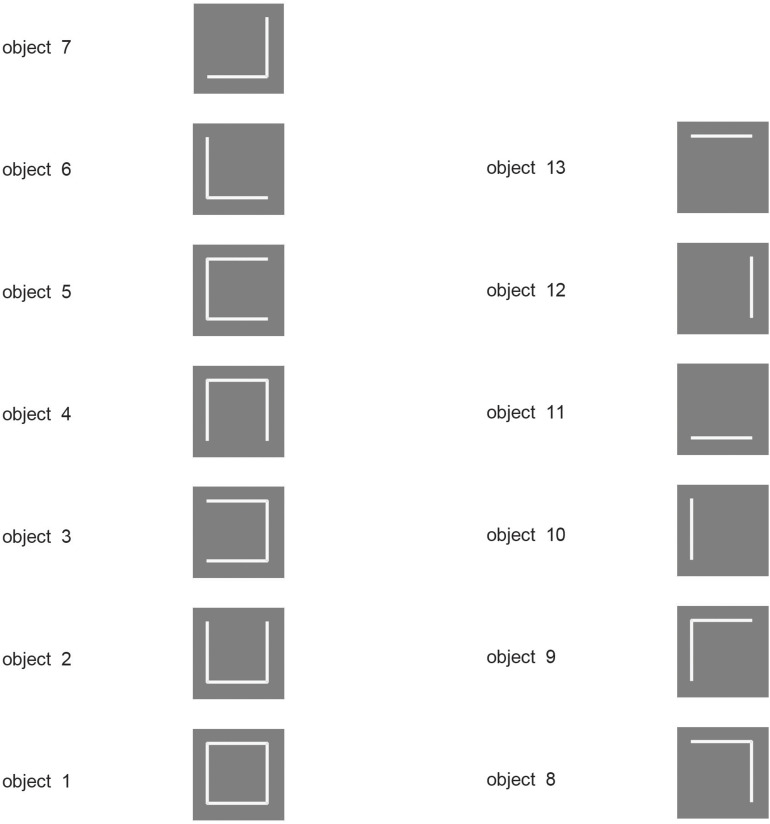
Encoding of information in intermediate Layers of VisNet. The 13 stimuli used to investigate independent coding of different feature combinations by different neurons in intermediate Layers of VisNet. Each of the 13 stimuli was a different feature, or feature combination with adjacent features, that was learned to be a different object by VisNet, demonstrating that VisNet can learn to represent objects as different even when they have overlapping features. Moreover, these feature combination neurons could be used by further combination in higher Layers of VisNet to represented more complex objects. (After Rolls and Mills, 2018).

To ensure that feature binding is accomplished with this architecture, VisNet was trained on stimuli that consisted of all possible combinations of the four lines that form a square (analogous to what is shown in Figure 2), and VisNet was able to learn correctly separate representations of all the resulting stimuli (Elliffe et al., 2002). The experiment also shows that VisNet can separate objects even though they are subsets or supersets formed from the same set of features (Elliffe et al., 2002). Thus feature binding operates well in VisNet, and later stages of VisNet can learn transform-invariant representations of each of these objects formed of different combinations of features in the correct spatial positions relative to each other.

Moreover, in a similar paradigm (Figure 2) it was shown that the feature combination neurons learned at intermediate Layers of VisNet can be used in the final Layer of VisNet as components of different objects (Rolls and Mills, 2018). This is important, for the use of feature combination neurons at intermediate stages for several different objects at the final stage is a key way that this architecture can use to represent many different objects, with a high capacity at the final stage, because the intermediate-stage representations are not just for a single object (Rolls and Mills, 2018). Part of the importance of this is that it shows that VisNet is not a look-up table.

Further, it was shown that if the intermediate Layers of VisNet are trained on feature combinations, then the final Layer of VisNet can learn about new objects that are formed from different combinations of what has been already learned in the intermediate Layers (Elliffe et al., 2002). In the real world, this potentially enables rapid learning of new objects in higher Layers of the system, because the early Layers will already have learned features that occur in the natural world.

The ways in which feature hierarchy networks are useful for solving the computational problems that arise in invariant visual object recognition are considered further by Rolls (2021a).

#### Operation in Cluttered Natural Environments, and With Partial Occlusion of Objects

Once trained on a set of objects, VisNet can recognize them in cluttered natural environments (Stringer and Rolls, 2000). The reason for this is that neurons are not tuned by learning to the cluttered background, so it does not interfere with the neuronal selectiveness which has been trained to the objects.

Further, once trained on a set of objects, partial occlusion of an object produces little impairment of performance (Stringer and Rolls, 2000), because the operation of the network is associative, and generalization occurs (Rolls, 2021a).

VisNet can also learn invariant representations of an object even if there are other objects in the scene, provided that the transforms of the object are presented close together in a sequence, with multiple other objects somewhere in the sequence (Stringer and Rolls, 2008). This is useful if the learning is about an object when sometimes other objects or backgrounds are present. The reason for this is that if the different transforms of one object are shown close together in the sequence, the invariance that will be learned is about those transforms of the object (Stringer and Rolls, 2008). This property is important for understanding that what is learned as invariant by VisNet is about the transforms that occur close together in time, and are therefore in the real world likely to be transforms of the same object. This was made clear in an experiment with morphological transforms described next.

In natural viewing conditions, the way in which lighting falls on objects can change their appearance, and training with the temporal trace learning rule can produce lighting transform-invariant representations (Rolls and Stringer, 2006a).

#### Invariance Over Morphological and 3D Transforms of Objects

**FIGURE 3:**
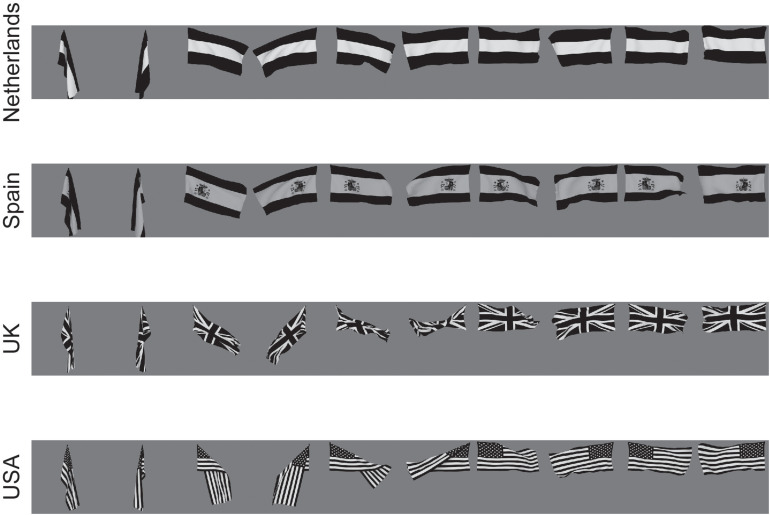
Deformation-invariant object recognition. The flag stimuli used to train VisNet to demonstrate deformation-invariant object recognition. Each flag is shown with different wind forces and rotations. Starting on the left with the first pair of images for each flag, both the 0 and 180° views are shown for a windspeed of 0; and each successive pair is shown for the wind force increased by 50 Blender units. Visnet learned to categorize these 4 flags as 4 different flags provided that the different deformations of each flag were shown close together in the temporal sequence during training, to make use of the trace learning rule. (After Webb and Rolls, 2014).

When a human is seen walking or sitting down, or standing up, one of these poses can be recognized independently of the individual, or the individual person can be recognized independently of the pose. The same applies to deforming objects. For example, for a flag that is seen deformed by the wind, either hanging languidly or blowing in the wind, the identity of the flag can usually be recognized independently of its deformation; or the deformation can be recognized independently of the identity of the flag [see Figure 3, which shows example of the images used in the investigation by Webb and Rolls (2014)].

Webb and Rolls (2014) hypothesized that the primate visual system can implement these different types of recognition by using temporo-spatial continuity as objects transform to guide learning. They hypothesized that pose can be learned when different people are successively seen in the same pose, or objects in the same deformation. They also hypothesized that representations of people that are independent of pose, and representations of objects that are independent of deformation and view, can be learned when individual people or objects are seen successively transforming through different poses or deformations and views (Webb and Rolls, 2014).

These hypotheses were tested with VisNet, and it was shown that pose-specific or deformation-specific representations were built that were invariant with respect to individual and view, if the statistics with which the inputs were presented included the same pose or deformation in temporal proximity (Webb and Rolls, 2014).

Further, it was shown that identity-specific representations were learned that were invariant with respect to pose or deformation and view, if the statistics with which the inputs were presented included the same person in different poses, or the same flag in different deformations, in temporal proximity (Webb and Rolls, 2014).

Webb and Rolls (2014) proposed that this is how pose-specific and pose-invariant, and deformation-specific and deformation-invariant, perceptual representations are built in the brain.

This illustrates an important principle, that information is present in the statistics of the inputs present in the world, and can be taken advantage of by slow learning of the type implemented in VisNet to learn different types of representation. This was powerfully illustrated in this investigation in that the functional architecture and stimuli were identical, and it was just the temporal statistics of the inputs that resulted in different types of representation being built (Webb and Rolls, 2014; Rolls, 2021a).

A similar principle applies to surface features on objects as the view of the object transforms: the appearance of the surface features transform. We showed that VisNet can learn view invariant transforms of 3D objects as they rotate into different views and their surface features transform (Stringer and Rolls, 2002).

#### Non-accidental Properties

**FIGURE 4:**
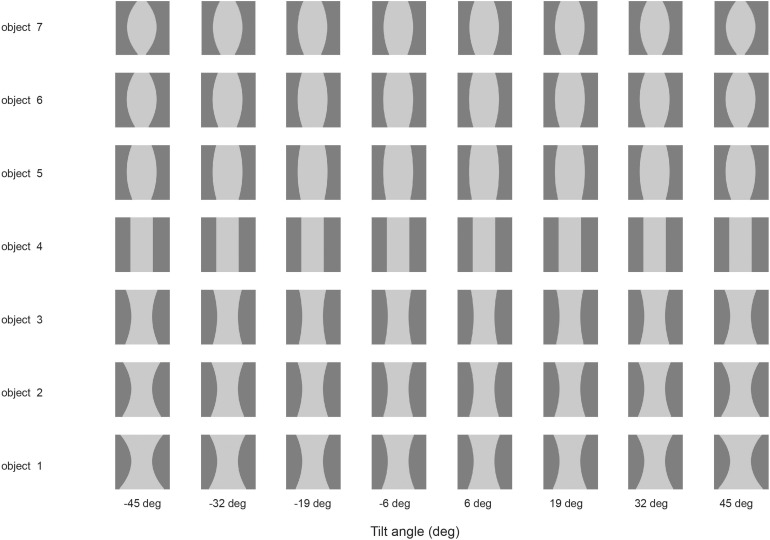
Learning non-accidental properties of objects. The stimuli used to investigate non-accidental properties (NAP) vs. metric properties (MP) of encoding in VisNet. Each object is shown as white on a gray background. Objects 1–3 all have the non-accidental property of concave edges. Objects 1–3 are different in their metric properties, the amount of curvature. Object 4 has the non-accidental property of parallel edges. Objects 5–7 have the non-accidental property of convex edges, and different metric properties from each other, the amount of the convexity. The vertical view of each object was at 0° of tilt, with the images at –6 and 6° of tilt illustrated. Different amounts of tilt of the top toward or away from the viewer are shown at the tilt angles indicated. Each object was thin, and was cut off near at the top and bottom of each object to ensure that any view of the top or bottom of the object did not appear, so that the type of curvature of the edges (concave, straight, or convex) was the main cue available. (After Rolls and Mills, 2018).

Some neurons in the visual system code for non-accidental properties of objects, such as convex vs. concave curvature vs. a straight edge (Vogels et al., 2001; Kim and Biederman, 2012). Non-accidental properties remain constant over view transforms, whereas the degree of curvature varies continuously with the transform (a metric property). We showed in VisNet how non-accidental properties of objects can be encoded as a result of self-organizing slow learning (Rolls and Mills, 2018), with the stimuli shown in Figure 4. Because of the trace learning rule, different transforms of objects produce different degrees of curvature, the metric property, but not different types of non-accidental property (such as concave vs. convex vs. straight), so neurons in VisNet learn to generalize over degree of curvature because a whole series occur close together in time while a particular object is being viewed, but not of non-accidental properties, which are different for different objects (Rolls and Mills, 2018).

The trace synaptic learning rule enables what is most persistent across time about an object to become learned as an invariant property, because that is how the statistics of real objects as they transform in the natural world behave (Rolls, 1992, 2021a; Wallis and Rolls, 1997). This is sometimes called slow learning and has been fruitfully followed up by a number of investigators (Wiskott and Sejnowski, 2002; Wyss et al., 2006; Franzius et al., 2007; Weghenkel and Wiskott, 2018), and may apply to the formation of complex cells in V1 (Matteucci and Zoccolan, 2020).

#### Receptive Fields of Inferior Temporal Cortex Neurons Shrink in Complex Natural Scenes, and Top-Down Attention Is Less Effective

The receptive fields of macaque inferior temporal cortex neurons are large (70° in diameter) with a blank background (which is how neurophysiology has classically been performed), but shrink to approximately 8° in radius (for a 5° stimulus) in complex natural scenes (Rolls et al., 2003). This has the great advantage that the output of the visual system in a complex natural world is primarily about the object at the fovea, so that subsequent stages of brain processing can represent the reward value of the object being looked at, and decide whether to perform actions toward that object (Rolls, 2016, 2021a). This greatly simplifies the neural computations that need to be performed, because the whole scene does not need to be processed at once, as in typical artificial vision systems, which thereby run into massive computational problems (Rolls, 2016, 2021a). Primates (including humans) have a fovea, and a greatly expanded cortical magnification factor for the fovea (Rolls and Cowey, 1970; Cowey and Rolls, 1975), to provide this functionality. Primates therefore use serial processing, by successive fixations on different parts of a scene, as necessary. An advantage of this functional architecture is that the coordinates for actions in space can be passed through the world, when the actions are toward a visually fixated object (Rolls, 2016, 2021a).

The mechanism for the shrinkage of the receptive fields of inferior temporal cortex neurons in complex natural scenes has been modeled by a network with greater cortical magnification for the fovea than for the periphery (Trappenberg et al., 2002). In a plain background, an object in the periphery can produce neuronal firing, because there is no competition from objects at the fovea. But when objects are at the fovea, they win the competition, because of the greater cortical magnification factor (Trappenberg et al., 2002).

Top-down attention, for example when an individual is searching a scene for a particular object, has a greater effect on neuronal responses for objects in a plain background than in a complex natural scene (Rolls et al., 2003). The same model accounts for this because when an object is at the fovea, the bottom-up visual inputs are relatively strong because of the large cortical magnification factor, and dominate the neuronal firing (Trappenberg et al., 2002).

#### Top-Down Attention for Objects or Spatial Locations

**FIGURE 5:**
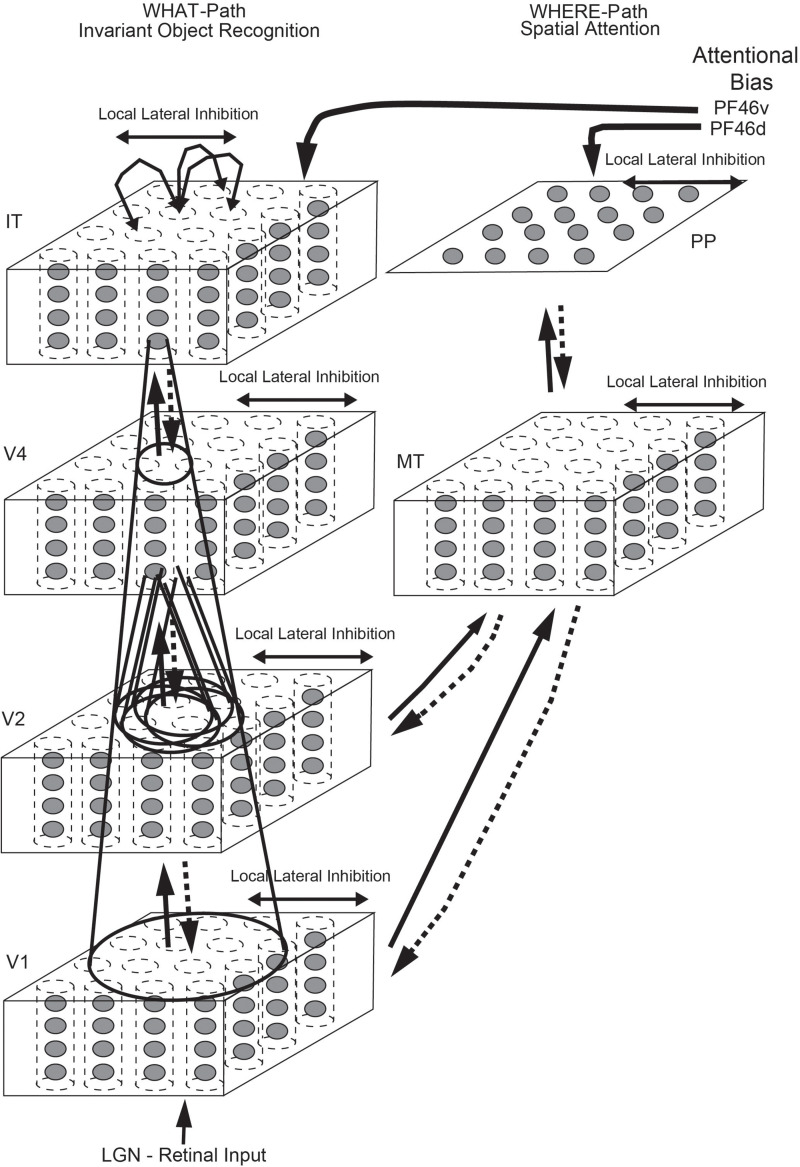
Cortical architecture for hierarchical and attention-based visual perception. The system has six modules organized so that they resemble the ventral visual stream **(Left)** and dorsal visual stream **(Right)** of the primate visual system. Information from the lateral geniculate (LGN) enters V1. The ventral visual stream leads through V2–V4 to the inferior temporal visual cortex (IT), and is mainly concerned with object recognition. The dorsal visual stream leads via areas such as MT into the posterior parietal cortex (PP), and is involved in this model in maintaining a spatial map of an object’s location. The solid lines with arrows between levels show the forward connections, and the dashed lines the top-down backprojections. Short-term memory systems in the prefrontal cortex (PF46) apply top-down attentional bias to the object (from PFv) or spatial processing (from OFd) streams. (After Deco and Rolls, 2004).

Top down attentional effects have also been investigated in a hierarchical VisNet-like network which incorporates a foveal cortical magnification factor and top-down projections with a dorsal visual stream so that attentional effects can be investigated, with the architecture illustrated in Figure 5 (Deco and Rolls, 2004). The architecture used the trace learning rule to achieve translation invariance. With this architecture, it was shown that the receptive fields were smaller in the complex natural scene than with a plain background; and that top-down selective attention (originating from the ventral prefrontal cortex PF46v in Figure 5) could act to increase the receptive field sizes of inferior temporal visual cortex (IT) neurons (Deco and Rolls, 2004). Investigations with a similar ‘what’/‘where’ architecture have shown how top-down attention to an object can have effects on the spatial representations; and how top-down attention to a location can have effects on which object is selected (Deco and Rolls, 2002, 2005; Rolls and Deco, 2002, 2006; Deco et al., 2004). (Many investigations with this architecture are described in *Computational Neuroscience of Vision* (Rolls and Deco, 2002), available for download^1^).

#### The Representation of Multiple Objects in a Scene With a Single Visual Fixation; And the Learning of Spatial Scenes by Hippocampal Spatial View Cells

When the neuronal representations of objects are distributed across a population of neurons, a problem arises about how multiple objects can be represented in a scene, because the distributed representations of different objects overlap, and it becomes difficult to determine whether one new object, or several separate objects, is present in the scene (Mozer, 1991), let alone the relative spatial positions of the objects in a scene. Yet humans are able to identify several different objects in a scene and their relative spatial locations even in short presentation times without eye movements (Biederman, 1972).

Aggelopoulos and Rolls (2005) investigated this in recordings from single inferior temporal visual cortex neurons with five objects simultaneously present in the neuronal receptive field. It was found that in this condition with simultaneously presented visual stimuli, all the neurons responded to their effective stimulus when it was at the fovea, and some neurons responded to their effective stimulus when it was at some but not other parafoveal locations 10 degrees from the fovea. This asymmetry demonstrates a way of encoding across a population of neurons the position of multiple objects in a scene, and their locations relative to the fovea. The positions of the object with respect to the fovea, and thus their spatial locations relative to other objects in the scene, can thus be encoded by the subset of asymmetric neurons that are firing (Aggelopoulos and Rolls, 2005).

Building on this foundation, it was shown in a unifying computational approach that representations of spatial scenes can be formed by adding an additional self-organizing Layer of processing beyond the inferior temporal visual cortex which learns and takes advantage of these asymmetries in the receptive fields in crowded scenes of inferior temporal cortex neurons (Rolls et al., 2008). Scenes consisting of a set of 4 objects presented simultaneously in 4 quadrants of a scene resulted in neurons in the fifth Layer learning representations that required the components of the scene to be in the correct fixed spatial relationship to each other (Rolls et al., 2008). This is one way in which it is proposed that spatial view cells, present in the hippocampus and parahippocampal gyrus (Rolls et al., 1989b, 1997a, 1998; Feigenbaum and Rolls, 1991; Rolls and O’Mara, 1995; Robertson et al., 1998; Georges-François et al., 1999; Rolls and Xiang, 2006; Rolls and Wirth, 2018; Rolls, 2021b) which receive from high order visual cortical areas (Epstein and Baker, 2019; Huang et al., 2021), learn to respond to scenes and indeed to particular locations in a scene (Rolls, 2021a, b).

#### Finding and Recognizing Objects and People in Natural Scenes: The Roles of the Dorsal and Ventral Visual Systems

When humans and other primates look at a visual scene, the eyes fixate on a succession of locations in a scene, and recognize the objects at each location. This greatly simplifies the task for the object recognition system, for instead of dealing with the whole scene as in traditional computer vision approaches, the brain processes just a small visually fixated region of a complex natural scene at any one time, and then the eyes move to another part of the scene. A neurophysiological mechanism that the brain uses to simplify the task of recognizing an object in complex natural scenes is (as described above) that the receptive fields of inferior temporal cortex neurons reduce from 70° in diameter when tested under classical neurophysiology conditions with a single stimulus on a blank screen, to as little as a radius of 8° (for a 5° stimulus) in a complex natural scene (Sheinberg and Logothetis, 2001; Rolls et al., 2003). When searching in a complex natural scene for an object, the high resolution fovea of the primate visual system is moved by successive fixations until the fovea comes within approximately 8° of the target, and then inferior temporal cortex neurons respond to the target object, and an action can be initiated toward the target object, for example to obtain a reward (Rolls et al., 2003). This experiment also provides evidence that the inferior temporal cortex neurons respond to the object being fixated with not only view, size, and rotation invariance, but also with some translation invariance, in that the eyes may be fixating 8° from the center of the object when the inferior temporal cortex neurons respond during visual search (Rolls et al., 2003).

The following question arises: how are the eyes guided in a complex natural scene to fixate close to what may be an object? The dorsal visual system deals with this by implementing a bottom-up saliency mechanism that can guide saccades to salient visual stimuli, using salient properties of the stimuli such as high contrast, color, and visual motion (Miller and Buschman, 2012). (Bottom-up refers to inputs reaching the visual system from the retina). A dorsal visual system region involved is the lateral intraparietal cortex (LIP), which contains saliency maps sensitive to strong sensory inputs (Arcizet et al., 2011). Highly salient, briefly flashed, visual stimuli capture the response of LIP neurons, and behavior (Goldberg et al., 2006).

We investigated computationally how a dorsal visual system bottom-up saliency mechanism could operate in conjunction with the ventral visual stream reaching the inferior temporal visual cortex to provide for invariant object recognition in natural scenes (Rolls and Webb, 2014). The hypothesis investigated was that the dorsal visual stream uses saliency to guide saccadic eye movements to salient stimuli in large parts of the visual field but cannot perform object recognition; and that the ventral visual stream performs invariant object recognition when the eyes are guided to be sufficiently close to the target object by the dorsal visual system. The experiments just described show that translation invariance of about 8° needs to be implemented in the ventral visual system for this mechanism because the eyes can be 8° from the target when it is recognized by inferior temporal cortex neurons, and an action is initiated, such as reaching to touch the object if it has been identified as a target object (Rolls et al., 2003; Aggelopoulos and Rolls, 2005). However, the ventral visual stream needs to implement not only this degree of translation invariance, but also size and view invariance to account for invariant object identification in natural scenes (Rolls, 2021a).

**FIGURE 6:**
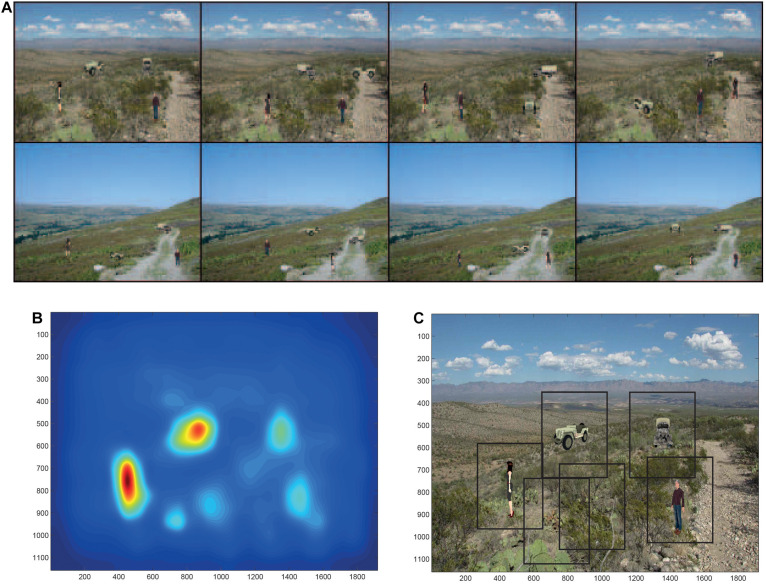
Finding and recognizing objects in natural scenes. **(A)** Eight of the twelve test scenes. Each scene has four objects, each shown in one of its 4 views. **(B)** The bottom-up saliency map generated by the GBVS code for one of the scenes. The highest levels in the saliency map are red, and the lowest blue. **(C)** Rectangles (384 pixels × 384 pixels) placed around each saliency peak in the scene for which the bottom-up saliency map is illustrated in **(B)**. (After Rolls and Webb, 2014).

To investigate how the dorsal and ventral visual systems may cooperate in object search and identification in complex natural scenes, we simulated a system with a dorsal visual system saliency map, and a ventral visual system model provided by VisNet that had to deal with translation invariance up to 8°, but also view invariance (Rolls and Webb, 2014). The dorsal visual system was simulated to provide a saliency map that would guide the locations to which visual fixations would occur. This was implemented with a bottom up saliency algorithm that adopts the Itti and Koch (2000) approach to visual saliency, and implements it by graph-based visual saliency (GBVS) algorithms (Harel et al., 2007). The basis for the saliency map consists of features such as high contrast edges, and the system knows nothing about objects, people, vehicles etc. This system performs well, that is similarly to humans, in many bottom-up saliency tasks (Harel et al., 2007). With the scenes illustrated in Figure 6A, the saliency map that was produced is illustrated in Figure 6B. The peaks in this saliency map were used as the sites of successive ‘fixations,’ at each of which a rectangle (of 384 pixels × 384 pixels) was placed, and was used as the input image to VisNet as illustrated in Figure 6C. VisNet had been trained on four views spaced 45° apart of each of the 4 objects/people, with a 25-location grid with a spacing of 16 pixels for translation invariance. We found that performance was reasonably good, in that the objects could be found in the complex natural scenes by the saliency mechanism, and identification of the object at the location to which the system had been guided by the saliency map was 90% correct where chance was 25% correct, for which object or person had been shown. That is, even when the fixation was not on the center of the object, performance was good. Moreover, the performance was good independently of the view of the person or object, showing that in VisNet both view and position invariance can be trained into the system using slow learning (Rolls and Webb, 2014). Further, the system also generalized reasonably to views between the training views which were 45° apart. Further, this good performance was obtained when inevitably what was extracted as it was close to the fovea included parts of the background scene within the rectangles illustrated in Figure 6C (Rolls and Webb, 2014).

This investigation elucidated how the brain may solve this major computational problem of recognition of multiple objects seen in different views in complex natural scenes, by moving the eyes to fixate close to objects in a natural scene using bottom-up saliency implemented in the dorsal visual system, and then performing object recognition successively for each of the fixated regions using the ventral visual system modeled to have both translation and view invariance in VisNet (Rolls and Webb, 2014). The research emphasizes that because the eyes do not find the center of objects based on saliency, then translation invariance as well as view, size etc. invariance needs to be implemented in the ventral visual system. The research showed how a model of invariant object recognition in the ventral visual system, VisNet, can perform the necessary combination of translation and view invariant recognition, and moreover can generalize between views of objects that are 45° apart during training, and can also generalize to intermediate locations when trained in a coarse training grid with the spacing between trained locations equivalent to 1–3° (Rolls and Webb, 2014; Rolls, 2021a).

#### Slow Learning in an Attractor Model of Invariant Object Recognition

VisNet uses a short-term memory trace learning rule in the feedforward connections of its competitive networks. An alternative architecture is to use an attractor network with a short-term memory trace learning rule in the recurrent collateral feedback connections. With this architecture it was shown that the number of objects *O* that can be stored and correctly retrieved is

O=kC/s

where *C* is the number of synapses on each neuron devoted to the recurrent collaterals from other neurons in the network, *s* is the number of transforms (e.g., views) of each object, and *k* is a factor that is in the region of 0.07–0.09 (Parga and Rolls, 1998). There is a heavy cost to be paid for large numbers of views *s*, and the approach of using the recurrent collaterals as an attractor network to perform transform invariant object recognition has not been pursued further. However, the recurrent collaterals could be useful to help to store categories of objects learned by using VisNet-like mechanisms. The object recurrent attractor would help to ‘clean up’ a somewhat ambiguous image into one or another object category, and indeed evidence for this has been found (Akrami et al., 2009). Further, the neocortex can be considered to perform competitive learning in a neuronal population in a brain area, supplemented by attractor or autoassociation properties endowed by the recurrent collateral connections (Rolls, 2016).

#### The Capacity of VisNet

Several factors that make a useful contribution to the number of objects that can be recognized by VisNet have been noted above. These factors include the use of sparse distributed representations, and the reuse of intermediate-Layer neurons as components of different objects represented at the final Layer. But how VisNet would scale up to provide a model of human visual object representations is a topic of interest. VisNet in quite a small form of 32 × 32 neurons in each of 4 Layers, and 200 synapses on to each neuron from the preceding Layer, is small compared to what is found in the neocortex. Cortical pyramidal cells often have in the order of 20,000 synapses per neuron, with perhaps 10,000 devoted to recurrent collateral inputs, perhaps 5,000 synapses to feedforward inputs that could be used for competitive learning, and perhaps 5,000 to backprojections ending in layer 1 (Rolls, 2016). The number of neurons in such a cortical module might be in the order of 100,000 (Rolls, 2016). Each such module would occupy a region of the cortical mantle with an area of a few mm^2^. An important property is that this connectivity is diluted, with the dilution in the order of perhaps 0.1, and that could help with capacity, as each neuron potentially receives a different combination of the afferents from the preceding cortical area. The ventral visual system could have tens to hundreds of such modules (Rolls, 2016).

With these factors in mind, it is difficult to know whether VisNet would scale up sufficiently to account for primate/human visual object recognition. What we do know at present is that a model of VisNet with the size specified above when trained on 50 real-world object images (Geusebroek et al., 2005) each with 9 views separated by 40° can represent the object from any view with 90% correct. (Chance is 2% correct). When tested with interpolated views each 20° from the nearest trained view, performance is 68% correct. These levels of performance are obtained with the Matlab-only implementation of VisNet that is made available with *Brain Computations: What and Where* (Rolls, 2021a) at https://www.oxcns.org.

#### Comparison of HMAX With VisNet

HMAX is an approach to invariant object recognition that builds on the hypothesis that not only translation invariance [as implemented by Fukushima (1980) in the Neocognitron], but also other invariances such as scale, rotation and even view, could be built into a feature hierarchy system (Riesenhuber and Poggio, 1999, 2000; Serre et al., 2007a, b). HMAX is a feature hierarchy network that uses alternate ‘simple or S cell’ and ‘complex or C cell’ Layers in a design analogous to Fukushima (1980). Each S cell Layer works by template matching based on the inputs received from the previous Layer. Each local patch of S cells is propagated laterally [that is, copied throughput the Layer, a property adopted also by deep convolutional neural networks (LeCun et al., 2015; Rajalingham et al., 2018), and of course completely biologically implausible (Rolls, 2016, 2021a)]. The function of each ‘C’ cell Layer is to provide some translation invariance over the features discovered in the preceding simple cell Layer, and operates by performing a MAX function on the inputs. A non-biologically plausible support vector machine (or least squares computation) performs classification of the representations of the final Layer into object classes. This is a supervised type of training, in which a target is provided from the outside world for each neuron in the classification Layer. The standard HMAX model (Riesenhuber and Poggio, 1999, 2000; Serre et al., 2007a, b; Mutch and Lowe, 2008) has no short-term memory trace slow learning synaptic modification rule. It is therefore interesting and informative to compare it with VisNet.

Robinson and Rolls (2015) compared the performance of HMAX and VisNet in order to help identify which principles of operation of these two models of the ventral visual system best account for the responses of inferior temporal cortex neurons. First, when trained with different views of a set of objects, HMAX performed very poorly because it has no mechanism to learn view invariance, i.e., that somewhat different images produced by a single object seen in different views are in fact of the same object. In contrast, VisNet learned this well, using its short-term memory trace learning rule to do this. Also, the final Layer of HMAX was found to have very non-selective and distributed representations, unlike those found in the brain (Robinson and Rolls, 2015).

Second, it was shown that VisNet neurons, like many neurons in the inferior temporal visual cortex (Perrett et al., 1982; Rolls et al., 1994), do not respond to images of faces in which the parts have been scrambled, and thus encode shape information, for which the spatial arrangements of the features is important. HMAX neurons responded to both the unscrambled and scrambled faces, indicating that the presence of low level visual features including texture may be relevant to HMAX performance, and not the spatial arrangements of the features and parts to form an object (Robinson and Rolls, 2015). Moreover, the VisNet neurons and inferior temporal cortex neurons encoded the identity of the unscrambled faces (Robinson and Rolls, 2015), and did this with sparse distributed representations, with well tuned neurons (Rolls and Tovee, 1995; Rolls et al., 1997c; Franco et al., 2007; Rolls and Treves, 2011; Rolls, 2021a). Further, the neurons in the last Layer of HMAX before the support vector machine had very distributed representations with poorly tuned neurons (Robinson and Rolls, 2015), quite unlike those in VisNet and the inferior temporal visual cortex just described.

**FIGURE 7:**
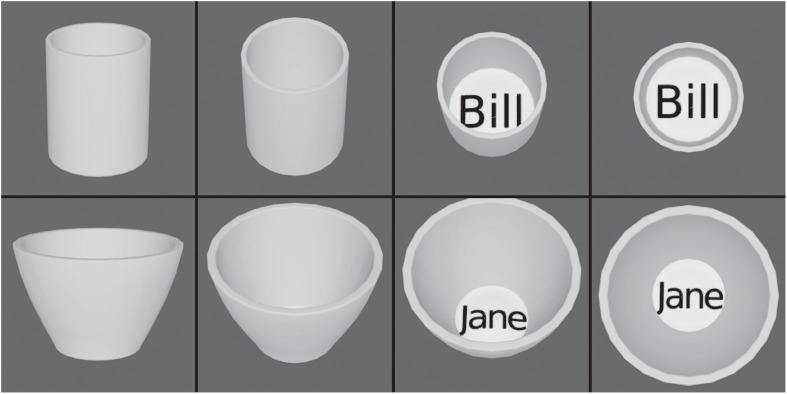
View invariant representations by VisNet but not by HMAX. The two objects, cups, each with four views. HMAX of Riesenhuber and Poggio (1999) fails to categorize these objects correctly, because, unlike VisNet, it has no slow learning mechanism to associate together different views of the same object. (After Robinson and Rolls, 2015).

Third, it was shown that VisNet can learn to recognize objects even when the view provided by the object changes catastrophically as it transforms, whereas HMAX has no learning mechanism in its S-C hierarchy that can perform such view-invariant learning (Robinson and Rolls, 2015). The objects used in the investigation with VisNet and HMAX are illustrated in Figure 7 (Robinson and Rolls, 2015). The two objects (two cups), each with four views, were made with Blender. VisNet was trained with all views of one object shown in random permuted sequence, then all views of the other object shown in random permuted sequence, to enable VisNet to learn with its temporal trace learning rule about the different images that occurring close together in time were likely to be different views of the same object. The performance of VisNet was 100% correct: it self-organized neurons in its Layer 4 that responded either to all views of one cup (labeled ‘Bill’) and to no views of the other cup (labeled ‘Jane’), or vice versa. HMAX neurons did not discriminate between the objects. Instead the HMAX neurons responded more to the images of each object that contained text. This strong influence of text rather than encoding for objects is consistent with the fact that HMAX is operating to a considerable extent as a set of image filters, the activity in which is much influenced by text regardless of which object it belongs to. HMAX has no mechanism within its S-C Layers that enables it to learn which input images belong to one object vs. another, whereas VisNet can solve this computational problem, by using temporal and spatial continuity present in the way that objects are viewed in a natural environment (Robinson and Rolls, 2015).

This highlights the importance of learning from the statistics produced by transforms of objects as they are viewed in the world, and shows how unsupervised slow learning can be successful (Rolls, 2021a). VisNet also shows how its type of learning can be performed without prejudging what is to be learned, and without providing a biologically implausible teacher for what the outputs of each neuron should be, which in contrast is assumed in HMAX and deep learning. Indeed, in deep learning with convolution networks the focus is still to categorize based on image properties (Rajalingham et al., 2018; Zhuang et al., 2021), rather than object properties that are revealed for example when objects transform in the world (Rolls, 2021a).

#### Comparison of Hierarchical Convolutional Deep Neural Networks With VisNet

A different approach has been to compare neuronal activity in visual cortical areas with the neurons that are learned in artificial models of object recognition such as hierarchical convolutional deep neural networks (HCNN) (Yamins and DiCarlo, 2016; Rajalingham et al., 2018). Convolution networks involve non-biologically plausible operations such as error backpropagation learning, and copying what has been set up in one part of a Layer to all other parts of the same Layer, which is also a non-local operation (LeCun et al., 2010, 2015; Bengio et al., 2017; Rolls, 2021a). They also require a teacher for each output neuron, which again is biologically implausible (Rolls, 2021a). The parameters of the hierarchical convolutional deep neural network are selected or trained until the neurons in the artificial neural network become similar to the responses of neurons found in the brain. The next step of the argument then seems to need some care. The argument that appears to be tempting (Yamins and DiCarlo, 2016; Rajalingham et al., 2018) is that because the neurons in the HCNN are similar to those in for example the inferior temporal visual cortex, the HCNN provides a model of how the computations are performed in the ventral visual system. But of course the model has been trained so that its neurons do appear similar to those of real neurons in the brain. So the similarity of the artificial and real neurons is not surprising. What would be surprising is if it were proposed that the HCNN is a model of how the ventral visual stream computes (Yamins and DiCarlo, 2016; Rajalingham et al., 2018), given that a HCNN with its non-local operation does not appear to be biologically plausible (Rolls, 2021a). VisNet, in contrast, utilizes only local information such as the presynaptic and postsynaptic firing rates and a slowly decaying trace of previous activity (that could be implemented by a local attractor network using the recurrent collateral connections), so is a biologically plausible approach to invariant visual object recognition (Rolls, 2021a).

Although progress has been made in unsupervised versions of deep convolutional neural networks trained with backpropagation of error (Zhuang et al., 2021), the network still relies on image features to discriminate objects, and therefore will have problems with learning view invariant object representations to solve problems such as that illustrated in Figure 7 in which different views of an object have different image properties (Robinson and Rolls, 2015). VisNet solves this and other aspects of invariant object recognition by using the statistics of the world captured by slow learning (Robinson and Rolls, 2015).

Another approach is to use unsupervised learning with a spike-timing dependent local synaptic learning rule, with a winner-take-all algorithm, and to transmit spikes, and this is reported to enable features to be extracted that are useful for classification (Ferre et al., 2018). This has been extended to deep convolutional neural networks for object recognition (Kheradpisheh et al., 2018).

### Unsupervised Learning for Object Recognition and Spatial View Cells Using the Spatial Statistics of Information From the World

The temporal continuity typical of objects as they transform in the natural world can be utilized by an associative learning rule with a short term memory trace to aid with the building of invariant object representations as set out above (Rolls, 2012, 2021a). However, there is another type of continuity that is present as most objects transform in the visual world, namely spatial continuity. We demonstrated that spatial continuity can provide a basis for a system to self-organize transform-invariant representations (Perry et al., 2006, 2010; Stringer et al., 2006). We introduced a new learning paradigm ‘continuous spatial transformation (CT) learning’ that can operate in neural systems by mapping similar spatial input patterns to the same postsynaptic neurons in a competitive learning network. While the inputs change through the space of possible continuous spatial transforms (e.g., translation, rotation, etc.), the active synapses are modified onto the set of postsynaptic neurons. Because other spatial transforms of the stimulus activate some of the same input neurons as previously learned exemplars, a common set of postsynaptic neurons is activated by the new transforms, and learning of the new active inputs onto the same postsynaptic neurons occurs.

**FIGURE 8:**
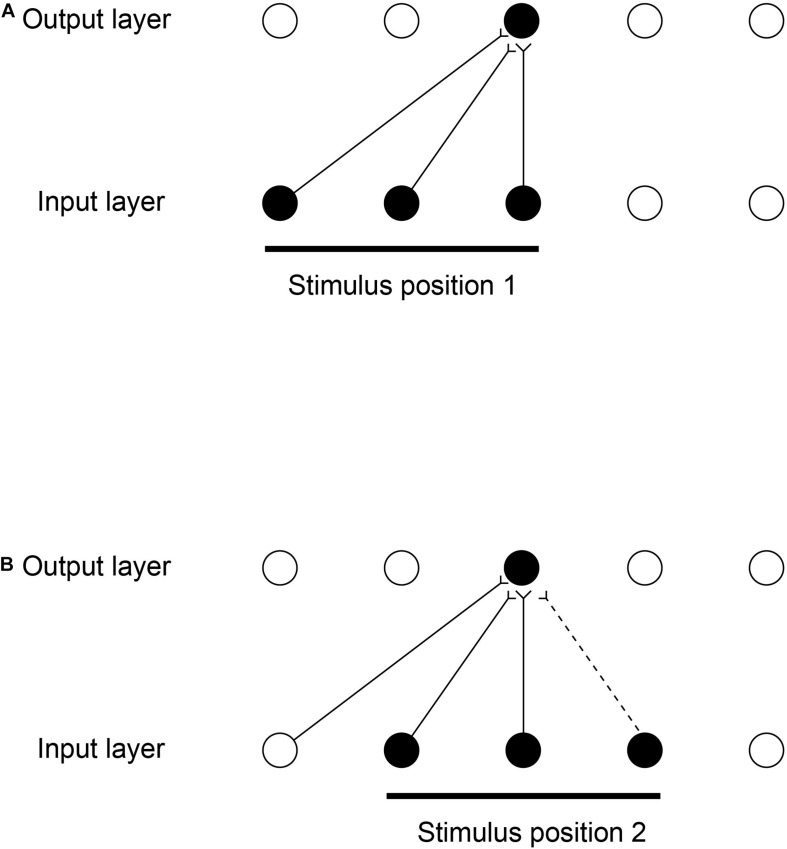
Continuous spatial transformation learning of transform-invariant visual representations of objects. This illustrates how continuous spatial transformation (CT) learning would operate in a network with forward synaptic connections between an input Layer of neurons and an output Layer. Initially the forward synaptic connection weights are set to random values. **(A)** The presentation of a stimulus to the network in position 1. Activation from the active (shaded black) input neurons is transmitted through the initially random forward connections to activate the neurons in the output Layer. The neuron shaded black in the output Layer wins the competition in the output Layer. The synaptic weights from the active input neurons to the active output neuron are then strengthened using an associative synaptic learning rule. **(B)** The situation after the stimulus is shifted by a small amount to a new partially overlapping position 2. Because some of the active input neurons are the same as those that were active when the stimulus was presented in position 1, the same output neuron is driven by these previously strengthened synaptic afferents to win the competition. The rightmost input neuron shown in black is activated by the stimulus in position 2, and was inactive when the stimulus was in position 1, now has its synaptic connection to the active output neuron strengthened (denoted by the dashed line). Thus the same neuron in the output Layer has learned to respond to the two input patterns that have vector elements that overlap. The process can be continued for subsequent shifts, provided that a sufficient proportion of input neurons is activated by each new shift to activate the same output neuron. (After Stringer et al., 2006).

The computational scheme is illustrated in Figure 8 (Perry et al., 2006, 2010; Stringer et al., 2006). While a visual image is presented at one location on the retina that activates neurons in Layer 1, a winning small set of neurons in Layer 2 associatively modify their afferent synaptic connections from Layer 1 to learn to respond to that image in that location. The same image shown later at nearby locations, will, because of spatial overlap, activate the same neurons in Layer 2 because some of the active afferents are identical with those when the image was in the first position. The key concept is that because these afferent connections have been strengthened sufficiently while the image is in the first location, then these afferent connections will activate the same neurons in Layer 2 when the image is shown in nearby overlapping locations. The result is that the same neurons in the output Layer learn to respond to inputs that have overlapping elements.

Figure 8 illustrates how the process can be continued for other shifts, provided that a sufficient proportion of input cells are activated by the individual shifts. The procedure is repeated throughout the network, both with the image moving across the retina, and hierarchically up through the network. Across the levels of the network, transform invariant (e.g., location invariant) representations of images are learned successfully, setting up the network to implement invariant object recognition. Similar CT learning can operate for other kinds of transformation, including transforms of view and size (Perry et al., 2006, 2010; Stringer et al., 2006).

VisNet can be trained with continuous spatial transformation (CT) learning to form view-invariant representations (Stringer et al., 2006). It was demonstrated that CT learning needs the training transforms to be spatially relatively close, so that spatial continuity is present in the training set; and that the order of stimulus presentation is not needed, with even interleaving with other objects possible during training, as spatial continuity rather than temporal continuity drives the self-organizing learning with the purely associative synaptic modification rule with no temporal trace short-term memory term.

This research on view invariant learning using CT with VisNet was extended to more complex 3D objects, which were also used in human psychophysical investigations. It was found that view invariant object learning can occur when spatial continuity (with no temporal continuity) is present in a training condition in which the images of different objects are interleaved (Perry et al., 2006). However, the human view invariance learning was better with sequential presentation of the images of each object, providing evidence that temporal continuity is an important factor in invariance learning in humans.

Continuous spatial transformation learning was further extended to translation invariance (Perry et al., 2010). It was shown that CT learning enables VisNet to learn translation invariant representations; that the transforms must be spatially close; that the temporal order of presentation of each transformed image during training is not crucial for CT learning of translation invariant representations; and that the number of transforms that can be learned is relatively large (Perry et al., 2010). CT learning can usefully be combined with temporal trace training as explored further (Spoerer et al., 2016).

Stringer et al. (2005); Rolls et al. (2008), and Rolls (2021a) proposed that the Gaussian spatial view fields of hippocampal spatial view cells enable representations of scenes to be learned due to associative learning driven by the overlap of the spatial view fields of different neurons as the individual looks from location to location in a viewed scene, including when the individual traverses through the environment. This forms a continuous attractor network that is effectively a representation of a scene and is formed by the overlaps of the spatial fields of neurons (Stringer et al., 2005; Rolls et al., 2008). This continuous attractor representation of a spatial scene facilitates navigation by enabling a trajectory through the continuous attractor of spatial view cells (Rolls, 2021b). Analogous mechanisms are proposed for place cell learning (Samsonovich and McNaughton, 1997; Stringer et al., 2002). These spatial continuous attractor networks are sometimes referred to as charts of an environment (Battaglia and Treves, 1998). It is noted here that this is in fact an example of the use of the spatial statistics of the world to build a representation, and is in fact CT (continuous spatial transform) learning. As shown above, these spatial charts can be built just by the overlap of spatial representations without slow learning (Perry et al., 2010), so the temporal order in which parts of a spatial scene are viewed is not a factor in how such navigational charts including spatial view representations of scenes are built. The use of the ‘spatial view cell charts’ of scenes for navigation is considered further in Section “Slow Learning and Coordinate Transforms for Spatial Functions Including Navigation.”

## Slow Learning for Object-Based Global Motion in the Dorsal Visual System

In the cortex in the anterior part of the superior temporal sulcus, which is a convergence zone for inputs from the ventral and dorsal visual systems (Rolls, 2021a), Hasselmo et al. (1989b) discovered some neurons that respond to object-based motion, for example to a head rotating clockwise but not anticlockwise. These neurons were discovered when the stimuli being shown to the macaque were real heads performing these movements. Other neurons responded to a head performing ventral flexion with respect to the body (i.e., the head of a standing person moving to look down). Systematic investigation with videos shown on a screen confirmed that the neurons respond independently of whether the head is upright or inverted, which reverses the optic flow across the retina (Hasselmo et al., 1989b). The movement that is encoded is thus with respect to the body, and is thus in object-based coordinates (Hasselmo et al., 1989b). It is proposed that neurons of this general type are important for natural social behavior, for some of these neurons respond to turning the head away, and also independently to closing the eyes, both of which break social contact and often occur together.

**FIGURE 9:**
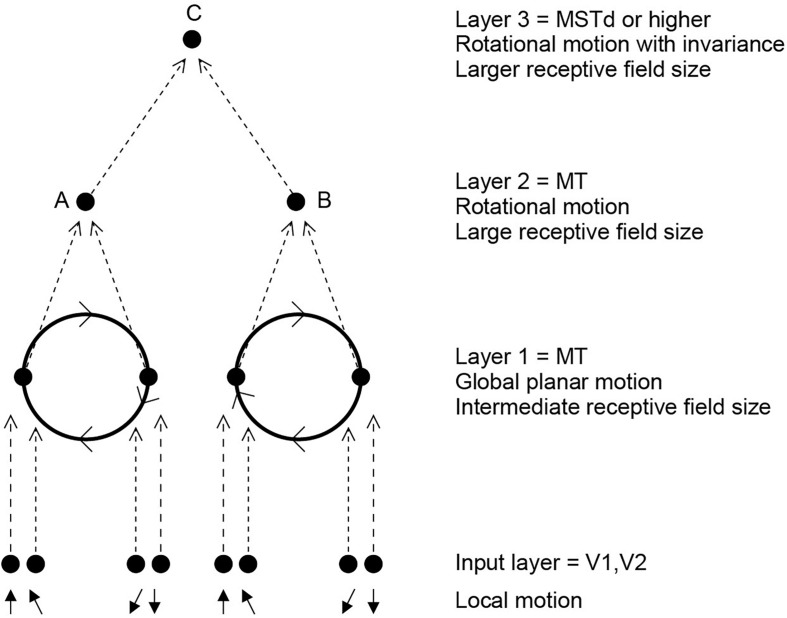
Invariant object-based global motion in the dorsal visual system. This shows two wheels at different locations in the visual field rotating in the same direction. One rotating wheel is presented at a time, and a representation is needed in the case illustrated that the rotating flow field produced by the wheel in either location is always clockwise. The local flow field in V1 and V2 is ambiguous about the direction of rotation of the two wheels, because of the small receptive field size. Rotation that is clockwise or counterclockwise can only be identified by a global flow computation, with larger receptive fields. The diagram shows how a network with stages like those found in the brain can solve the problem to produce position invariant global motion-sensitive neurons by Layer 3. The computation involved is convergence from stage to stage as illustrated, combined with a short-term memory trace synaptic learning rule to help the network learn that it is the same wheel rotating in the same direction as it moves across the visual field during training (during development). This is the computational architecture of VisNet. It was demonstrated that VisNet can learn translation invariant representations of these types of object-based motion, by substituting the normal Gabor filters as the input neurons in the input Layer corresponding to V1 with local optic flow motion neurons also present in V1. (After Rolls and Stringer, 2006b).

In a unifying hypothesis with the design of the ventral cortical visual system about how this might be computed, Rolls and Stringer (2006b) proposed that the dorsal visual system uses a hierarchical feedforward network architecture (V1, V2, MT, MSTd, and parietal cortex) with training of the synaptic connections with a short-term memory trace associative synaptic modification rule to compute what is invariant at each stage. Figure 9 illustrates the principle. It was demonstrated with simulations that the proposal is feasible computationally, in that invariant representations of the motion flow fields produced by objects self-organize in the higher Layers of the architecture. The computational architecture produces invariant representations of the motion flow fields produced by global in-plane motion of an object, in-plane rotational motion, and receding vs. looming of the object. Invariant representations of object-based rotation about a principal axis, of the type discovered by Hasselmo et al. (1989b), were also produced in the model (Rolls and Stringer, 2006b).

We thus proposed that the dorsal and ventral visual systems may share some unifying computational principles (Rolls and Stringer, 2006b). In fact, the simulations used a standard version of VisNet, except that instead of using oriented bar (/Gabor filter) receptive fields as the input to the first Layer of VisNet, local motion flow fields provided the inputs.

The interesting and quite new principle is that some of the same mechanisms including trace rule learning and hierarchical organization that are used in the ventral visual system to compute invariant representations of stationary objects may also be used in the dorsal visual system to compute representations of the global motion of a moving object. This may well be an example of a principle of cortical operation, the re-use of the same principles of cortical operation for different computations in different cortical areas (Rolls, 2016, 2021a).

## Slow Learning and Coordinate Transforms for Spatial Functions Including Navigation

### Slow Competitive Network Learning Can Help to Convert Entorhinal Cortex Grid Cells to Dentate/Hippocampal Place Cells

Grid cells in the medial entorhinal cortex are activated when a rodent is located at any of the vertices of a spatial grid of equilateral triangles covering the environment (Giocomo et al., 2011; Moser et al., 2015). However, cells in the dentate gyrus and hippocampus of the rodent typically display place fields, where individual cells are active over only a single portion of the space (O’Keefe, 1979; Jung and McNaughton, 1993; Leutgeb et al., 2007; Moser et al., 2015). In a model of the hippocampus, we have shown that the connectivity from the entorhinal cortex to the dentate granule cells could allow the dentate granule cells to operate as a competitive network to recode their inputs to produce sparse orthogonal representations, and this includes spatial pattern separation. We further showed that the same computational hypothesis can account for the mapping of entorhinal cortex grid cells to dentate place cells (Rolls et al., 2006b). It was shown that the learning in the competitive network is an important part of the way in which the mapping can be achieved (Rolls et al., 2006b). This approach has received support (Si and Treves, 2009). But we further showed that incorporation of a short term memory trace into the associative learning to implement slow learning can help to produce the relatively broad place fields found in the hippocampus (Rolls et al., 2006b).

It is now proposed that this same slow learning may help to account for the shape of place fields, which become distorted if there is an obstruction in the environment (Muller and Kubie, 1987). It is proposed that because the places on different sides of a barrier are not encountered close together in time, the place fields lose their continuity at the barrier, and stop at the barrier, because the spatial locations on each side of the barrier are not encountered close together in time, and so do not enable the slow learning to make the fields continuous across the barrier. This slow learning approach takes time into account, as does the reinforcement learning approach (Stachenfeld et al., 2017).

### Spatial View Cells

There is much evidence that the rodent hippocampus with its place cells is involved in memory and navigation (O’Keefe and Dostrovsky, 1971; O’Keefe and Nadel, 1978; O’Keefe, 1979; McNaughton et al., 1983, 2006; Burgess and O’Keefe, 1996; Morris et al., 2003; Takeuchi et al., 2014; Edvardsen et al., 2020). When we recorded in the macaque hippocampus, we found some place cells (Rolls and O’Mara, 1995), but very interestingly, many other neurons responded to where the monkey was looking in space (Rolls et al., 1989b; Feigenbaum and Rolls, 1991; Rolls and O’Mara, 1995). Bruce McNaughton suggested that the monkey should be allowed to locomote, and then investigate whether the spatial view cells might alter their properties, or place cells might become more evident. Rolls et al., 1997a devised a system that enabled the monkey to run quite naturally around the lab while recordings were made of hippocampal neuronal activity in a much richer environment, the rich environment of a large open laboratory. Careful measurement of the place, head direction, and eye position of the monkey during this locomotion showed that the spatial view neurons encoded most information about where the monkey was looking in allocentric space, and not about place, head direction, or eye position (Rolls et al., 1997a, 1998; Robertson et al., 1998; Georges-François et al., 1999; Rolls and Wirth, 2018). The much visually richer open lab environment also increased the proportion of spatial view cells, compared to the cue-controlled environment used previously (Rolls and O’Mara, 1995).

A key discovery was that these spatial view cells are updated in the dark by self-motion. For example, a spatial view cell in the dark, with curtains also blocking any view of the spatial scene, responds when the macaque looks toward the spatial view location where it responded in the light, and not when the macaque looks elsewhere. The spatial view field was thus similar in the light and the dark for many of these neurons (Robertson et al., 1998). This continued for only a few minutes, after which the spatial view field drifted, as the idiothetic (self-motion) update requires path integration involving a memory system (Robertson et al., 1998). This idiothetic update is potentially very useful in the natural world, for if a spatial view is obscured for a short time by an obstruction, then the spatial view system can continually update the locations in space to maintain navigation for short periods while the spatial view is obscured (Rolls, 2021b).

Thus this research involving foraging in an open lab visually rich environment enabled us to reveal many properties of spatial view cells, and further to show that they are involved in memory of where objects (Rolls et al., 2005; Rolls and Xiang, 2006) and rewards (Rolls and Xiang, 2005) are in viewed space. Further, it is now proposed that these spatial view cells are important not only in episodic memory (Kesner and Rolls, 2015; Rolls, 2018), but also in navigation (Rolls and Wirth, 2018; Rolls, 2021b). Indeed, the theory is that spatial view cells are very well suited to navigation in primates including humans, for they offer a natural way to navigate from landmark to landmark without explicit geometrical calculations in a Euclidean space (Rolls, 2021b). The mechanism is much simpler than the navigational systems proposed for rodents based on place cells in the hippocampus and grid cells in the entorhinal cortex involving maps of Euclidean space (O’Keefe, 1979; Burgess and O’Keefe, 1996; Bicanski and Burgess, 2018; Edvardsen et al., 2020).

The underlying mechanisms for navigation using spatial view cells in primates including humans, and how slow learning may be involved, are considered in Section “Slow Learning Combined With Gain Modulation for Learning Coordinate Transforms in the Dorsal Visual System Through to the Parietal Cortex, for Use in Hippocampal Navigation.”

### Slow Learning Combined With Gain Modulation for Learning Coordinate Transforms in the Dorsal Visual System Through to the Parietal Cortex, for Use in Hippocampal Navigation

A problem arises with navigation involving hippocampal spatial view cells and approach to a sequence of viewed landmarks if during navigation the landmarks are temporarily obscured. In this situation, idiothetic update, that is update based on self-motion, of spatial view cells (Robertson et al., 1998), can be used, it is proposed. This enables the location in the scene produced by idiothetic update to produce hippocampal spatial view firing when the monkey is looking toward the obscured view (Robertson et al., 1998), and could therefore be used to guide navigation toward the location in allocentric space where the relevant spatial view cells fire (Rolls, 2021b).

**FIGURE 10:**
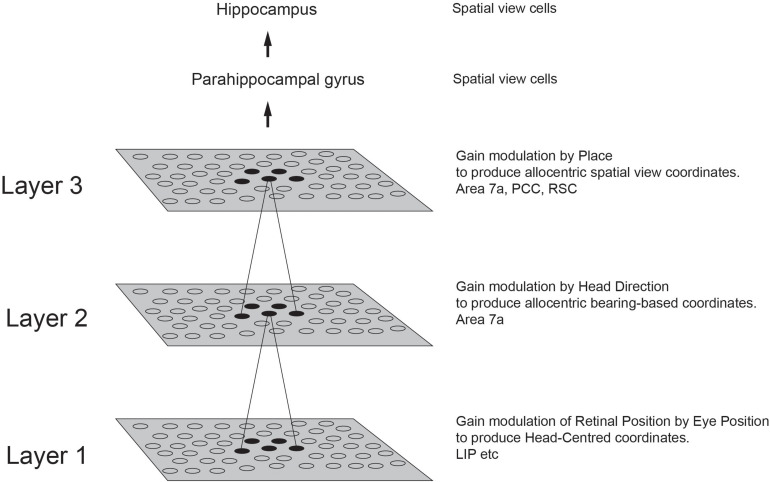
Coordinate transforms in the primate dorsal visual system. Three stages of coordinate transforms that take place at different levels of the primate dorsal visual system are shown. At each stage the coordinate transform is performed by gain modulation of the receptive field by an appropriate modulator, that is usefully combined with slow learning of the type implemented in VisNet which helps the same neurons at a particular stage to develop what are effectively representations that become independent of the modulating signal. In Layer 1 gain modulation by eye position combined with slow learning enables neurons to develop representations in head-centered coordinates that are invariant with respect to retinal and eye position. In Layer 2 gain modulation by head direction combined with slow learning enables neurons to develop representations in allocentric bearing to a stimulus such as a landmark coordinates that are invariant with respect to head direction. In Layer 3 gain modulation by the place where the individual is located combined with slow learning enables neurons to develop representations of a stimulus such as a landmark that are in allocentric spatial view coordinates with invariance with respect to where the individual is located. The diagram shows the architecture of the VisNetCT model in which gain modulation combined with short-term memory trace associative learning was shown to implement these transforms (Rolls, 2020). Each neuron in a Layer (or cortical area in the hierarchy) receives from neurons in a small region of the preceding Layer. It is proposed that idiothetic update through this dorsal visual cortical stream is used for idiothetic update of hippocampal spatial view cells useful for navigation when the environment may not be visible for short periods (Rolls, 2020, 2021b). PCC, posterior cingulate cortex; RSC, retrosplenial cortex.

The mechanism for idiothetic update of spatial view cells in primates needs to take into account eye position, as well as head direction and the place where the individual is located. Consistent with the neurophysiology of the primate dorsal visual system (Snyder et al., 1998; Dean and Platt, 2006), it is proposed that the coordinate transforms take place across a series of stages of the dorsal visual system hierarchy through the parietal cortex and thence via the retrosplenial cingulate cortex and posterior cingulate cortex to the hippocampal system via the parahippocampal gyrus (Rolls, 2020) (Figure 10).

This hierarchy transforms from egocentric representations to ‘allocentric bearing to a landmark’ and then to allocentric spatial view representations, with the brain regions for each stage indicated in Figure 10 (Rolls, 2020). The system starts with representations in retinal coordinates, and transforms these into head-based egocentric representations in LIP and VIP using gain modulation by eye position (Salinas and Abbott, 2001; Rolls, 2020). Then the head-centered representation is transformed into an ‘allocentric bearing to a landmark’ representation in areas such as parietal cortex 7a (Snyder et al., 1998) and posterior cingulate cortex (Dean and Platt, 2006) using gain modulation by head direction (Rolls, 2020). These neurons fire when a macaque views a stimulus in allocentric space (Snyder et al., 1998; Dean and Platt, 2006; Rolls, 2020). Then the ‘allocentric bearing to a landmark’ representation is transformed into an allocentric spatial view representation by gain modulation using translation of the individual to different places (Rolls, 2020). This builds a representation in the same spatial coordinates used in the primate hippocampus and parahippocampal gyrus, namely allocentric spatial view that represents a location in allocentric space ‘out there’, independently of the exact place where the animal is located, as well as its head direction and eye position (Rolls et al., 1997a, 1998; Robertson et al., 1998; Georges-François et al., 1999). This type of representation is ideal for the episodic memory functions of the primate hippocampus, for it enables memories to be formed of where in allocentric space an object or person was seen. Because the memory is independent of the exact place where the individual is located, if the same location is seen from a different place, the hippocampal memory system will correctly recall the object or person that was at that location (Rolls, 2018, 2021a). Similarly, if the object or person is the recall cue, the location in allocentric space where they were seen can be recalled from the CA3 network in the hippocampus, and that memory is suitable for navigation to that location, because it does not depend on the place where the animal is, which would be very restrictive indeed in a navigation or memory system (Rolls, 2020).

The mechanism proposed at each stage is gain modulation, but supplemented by trace rule slow learning as this greatly helps to improve the coordinate transform by reducing effects of imprecision which otherwise accumulate through the multi-stage system shown in Figure 10 (Rolls, 2020). It is well established that starting with retinal coordinates, gain modulation by eye position can transform the representation into head-based egocentric representations in LIP and VIP (Pouget and Sejnowski, 1997; Salinas and Abbott, 2001; Salinas and Sejnowski, 2001). This general mechanism was extended to two further stages in the dorsal visual system, as shown in Figure 10 (Rolls, 2020). Now, these types of coordinate transform are in effect a form of invariance learning. First the representation becomes invariant with respect to eye position, then with respect to head direction, and then with respect to place, to produce the idiothetic update of spatial view cells (Figure 10) (Rolls, 2020). Rolls reasoned that therefore slow learning using the short-term memory trace learning rule should help the coordinate transform learning, by enabling the system to produce the same output in for example head direction coordinates over a whole set of different eye positions occurring while a visual stimulus remained at the same location in space relative to the head. This invariance slow learning mechanism was shown to greatly improve the performance in a computational model of the processes, and helped in the formation of spatial view cells that are invariant with respect to eye position, head direction, and the place of the individual (Rolls, 2020). Spatial view cells may be very useful in primates including humans not only in episodic memory characterized by associations between objects and locations viewed in space (Kesner and Rolls, 2015; Rolls, 2018, 2021a), but also in navigation toward a sequence of viewed locations using spatial view cells (Rolls, 2021a, b).

This therefore provides another interesting example of how the statistics of the world, in the example just given a constant location in the world that is being looked at (a ‘spatial view’) invariantly with respect to eye position, head direction, or the place where the individual is, help slow unsupervised learning to produce behavior that is of great adaptive value in the natural world for episodic memory and for navigation (Rolls, 2020, 2021a).

The overall theory of how allocentric spatial view cells are formed in the first place, and can then be idiothetically updated in the way just described by self-motion inputs which must necessarily be converted into corresponding allocentric coordinates, is in brief as follows, with more detail elsewhere (Rolls, 2021a, b). The proposal is that hippocampal spatial view cells are driven by parts of scenes which may contain features represented in the ventral visual system. Different spatial view cells are linked together to form a scene representation in a continuous attractor network (Stringer et al., 2005). In a continuous attractor network the synaptic connections are strengthened between neurons that are nearby in the space, because they have coactive firing due to the approximately Gaussian shape of their overlapping spatial view fields. This sets up a continuous map of space in which adjacent points in the space are joined by their learned co-active firing due to their nearness in the viewed space, as shown for spatial view cells (de Araujo et al., 2001; Rolls and Stringer, 2005; Stringer et al., 2005; Rolls, 2016, 2021a). This enables the space to be read out continuously and sequentially, as a bubble of neural activity traverses the space (Rolls, 2021a). These spatial view cells to be useful for memory of where an object or person is in the spatial environment, and for navigation, and for imagery, and for the Art of Memory (Rolls, 2017), need to be invariant with respect to the exact position on the retina, eye position, head direction, and place where the viewer is located [and they are, as described above and elsewhere (Georges-François et al., 1999; Rolls, 2021b)]. The mechanism just described with the primate fovea which provides a locally Gaussian spatial view of the world enables the appropriate spatial scene representations to be formed, which do not depend on where the viewer is etc. because the representations are built just by the nearness of locations in a scene. These locations are linked in the correct spatial arrangement by associative synaptic learning of coactive spatial view cells with overlapping spatial fields as the viewer looks at different parts of the scene. The problem arises when the scene is temporarily obscured – can the part of the scene that the viewer is looking toward be updated by self-motion, to enable scene location-object memory recall, and navigation, to be performed? That is what is achieved by the dorsal visual system coordinate transform mechanisms described above, which utilize slow learning and gain modulation as the underlying mechanisms, which can be repeated stage after stage as illustrated in Figure 4 (Rolls, 2020).

The situation may be different in rodents, which do not have a fovea nor a highly developed dorsal visual system for eye movement control nor a posterior cingulate cortex, and which may rely more on place-based navigation rather than spatial view cell navigation (Rolls, 2021a, b). Slow learning may also be useful in the learning of place cell representations (Franzius et al., 2007; Schonfeld and Wiskott, 2015).

## Discussion and Conclusion

An interesting issue to consider is that in the ventral visual cortical stream, in the progression from V1 to V2 to V4 to posterior (TEO) and then to anterior inferior temporal visual cortex (TE), pyramidal cell basal dendrites cover a larger area of cortex, have a greater dendritic length, have a greater spine density, and have recurrent collateral connections that spread approximately over a region that is as large as the areal spread of the dendrites (Lund et al., 1993; Fujita and Fujita, 1996; Elston and Rosa, 1997, 1998; Elston, 2002, 2007; Jacobs and Scheibel, 2002; Elston and Fujita, 2014; Luebke, 2017; Oga et al., 2017). The average number of spines (each reflecting an excitatory synaptic input) on the basal dendrites of macaque layer 3 cortical pyramidal cells is in the order of 640 in V1, 1,139 in V2, 2,429 in V4, 4,812 in TEO, and 7,400 in TE (Elston and Rosa, 1998; Elston, 2007). (These numbers are likely to be approximately doubled by the backprojection inputs from higher cortical areas that terminate especially but not exclusively in the superficial cortical layers especially layer 1 of the neocortex (Abeles, 1991; Markov et al., 2013, 2014a, b; Markov and Kennedy, 2013; Rolls, 2016).

In relation to the computational processes taking place in the ventral cortical visual stream, the relatively small dendritic area and numbers of spines in early stages such as V1 and V2 are hypothesized to relate to the importance of maintaining high spatial resolution for individual neurons. In VisNet in Layer 1 which corresponds to V2 (Figure 1), this allows feature combination neurons to be formed that reflect the exact positions of the two features, so that e.g., ‘T’ can be distinguished from ‘L.’ (In this example the two features are a horizontal and vertical line). This must be performed before translation invariance is computed, and before the spatial position is less precisely represented, for otherwise spatial feature combination learning in which a neuron becomes sensitive to the exact spatial relation of the features, and hence the ability to distinguish different objects with similar features, would be impaired. For this computational reason, the learning allowed in Layer 1 of VisNet is purely associative, with no slow temporal trace, to minimize translation invariance learning in Layer 1. Further, given that temporal trace slow learning may be facilitated in part by the short-term memory implemented by local attractor networks utilizing the recurrent collateral connections between cortical pyramidal cells that terminate especially on the basal dendrites, the relatively small numbers of spines on the basal dendrites is also probably related to no need at early stages of visual processing for the neuronal activity to be maintained in short-term memory for short periods of a second or two while different transforms of an object may be presented in line with the natural statistics of the viewed world.

In contrast, at higher levels of VisNet (Layers 2–4 corresponding to V4, posterior inferior temporal cortex TEO, and to anterior inferior temporal cortex TE, see Figure 1), the aim is to extend the spatial receptive fields of neurons so that they can receive all the information needed to encode a given object by neuronal firing for all the different transforms of the object that are possible, including translation, size, and view. Each neuron by the final Layer (4) of VisNet and the real visual system in the brain must be able to receive information from across much of the visual field that can potentially be stimulated by all possible transforms of a given object, and that is achieved by the convergent multistage feedforward architecture illustrated in Figure 1 with therefore large dendritic trees by the end of the visual system. The premium here is on receiving many inputs from a wide local region of cortex that might be involved in responding to *any* transform of a single object. Further, given that attractor networks are likely to be implemented in the neocortex by the local recurrent collateral synapses between pyramidal cells that are likely to terminate mainly on the basal dendrites, the large numbers of synapses on each neuron in higher visual cortical areas may be very helpful for implementation of the short-term memory trace rule that is used for the slow learning, which may need to be robustly maintained for periods as long as one to a few seconds while different transforms of a given object are being seen. For this attractor system to work, large numbers of synapses devoted to the recurrent collateral excitatory connections are needed, because the number of such synapses on each neuron sets the number of different short-term memories in the attractor network in which a neuron can participate (Hopfield, 1982; Treves, 1991; Treves and Rolls, 1991; Rolls, 2021a). The number of recurrent collateral synapses on each neuron needs to be large because of the sparse distributed encoding used in the neocortex in which each neuron may participate in the encoding of many different objects, with different firing rates to each object (Rolls and Tovee, 1995; Franco et al., 2007; Rolls and Treves, 2011; Rolls, 2021a). Consistent with a short-term memory trace implemented in the inferior temporal visual cortex, these neurons continue to fire for often 1 s after the termination of a stimulus (unless a backward mask is applied, which provides evidence that the maintenance is an active process) (Rolls and Tovee, 1994; Rolls et al., 1999; Rolls, 2003).

The tutorial version of VisNet (Rolls, 2021a) allows parameters such as the radius in the preceding Layer from which inputs are received, the number of synapses per neuron for the inputs in each Layer, and whether a purely associative or instead a memory trace learning rule is used in each Layer, as these parameters are important in the theory of the learning of transform invariant representations in the ventral visual system and in the operation of VisNet.

The aim of the research described here has been to better understand how computations are performed by the brain, with special reference to how transform-invariant representations useful for vision are formed in the brain. The focus has therefore been on biologically plausible mechanisms, and further details of these are provided in *Brain Computations: What and How* (Rolls, 2021a).

However, what has been elucidated here has implications for training artificial neural networks. A key implication is that it can be helpful to utilize information available in the temporal and spatial statistics of the inputs, which as shown here can provide important information for the learning of transform-invariant representations that are useful in the natural world, or for that matter elsewhere. This is essentially a form of unsupervised learning, guided by the statistics of the inputs. It is unsupervised in the sense that there is no teacher for each output neuron as in deep convolution networks (LeCun et al., 2010, 2015) [which is biologically implausible (Rolls, 2016, 2021a)]. Nor does the training described here use reinforcement learning (Sutton and Barto, 1998; Schultz, 2016; O’Doherty et al., 2017).

Another aspect of the type of training described here is that it is systematic, with different views of the same object being presented, as typically occurs when objects are viewed in the natural world. In contrast, for learning with deep convolution networks, typically thousands of objects are used in ‘brute force’ training, with no systematic sets of transforms of the same objects to help the learning of transform-invariant representations. Another property of the brain is that it is able to perform its computations for invariances in networks with just 4 or 5 Layers (see Figure 1). Part of the reason for this is to maximize processing speed, and minimize computation and reaction time (Rolls, 2016, 2021a), but it does show that networks with one hundred or more Layers are not needed to solve the computations involved in transform-invariant object recognition.

What is described here and elsewhere (Rolls, 2021a) may thus it is hoped be useful for developing better artificial neural networks and artificial intelligence. For example, convolutional neural networks are typically trained on very large numbers of single training image exemplars (snapshots) of the classes to be learned, and can fail if a few pixels are altered, implying that they learn pixel-level representations. It is proposed here that training such networks with different transforms of objects would much better enable transform-invariant shape-based representations to be learned, leading to much more powerful performance. Potential limitations of current deep learning methods have been also been noted by others (Plebe and Grasso, 2019; Sejnowski, 2020).

## Author Contributions

The author confirms being the sole contributor of this work and has approved it for publication.

## Conflict of Interest

The author declares that the research was conducted in the absence of any commercial or financial relationships that could be construed as a potential conflict of interest.

## Footnotes

1 https://www.oxcns.org
